# Genome Sequence of *Rhizobium jaguaris* CCGE525^T^, a Strain Isolated from *Calliandra grandiflora* Nodules from a Rain Forest in Mexico

**DOI:** 10.1128/MRA.01584-18

**Published:** 2019-02-28

**Authors:** Luis E. Servín-Garcidueñas, Gabriela Guerrero, Marco A. Rogel-Hernández, Esperanza Martínez-Romero

**Affiliations:** a Laboratorio de Microbiómica, Escuela Nacional de Estudios Superiores Unidad Morelia, Universidad Nacional Autónoma de México (UNAM), Morelia, Michoacan, Mexico; b Centro de Ciencias Genómicas, Departamento de Ecología Genómica, Universidad Nacional Autónoma de México (UNAM), Cuernavaca, Morelos, Mexico; University of Delaware

## Abstract

We present the genome sequence of Rhizobium jaguaris CCGE525^T^, a nitrogen-fixing bacterium isolated from nodules of Calliandra grandiflora. CCGE525^T^ belongs to Rhizobium tropici group A, represents the symbiovar calliandrae, and forms nitrogen-fixing nodules in Phaseolus vulgaris. Genome-based metrics and phylogenomic approaches support Rhizobium jaguaris as a novel species.

## Abstract (precis)

We present the genome sequence of Rhizobium jaguaris CCGE525^T^, a nitrogen-fixing bacterium isolated from nodules of Calliandra grandiflora. CCGE525^T^ belongs to Rhizobium tropici group A, represents the symbiovar calliandrae, and forms nitrogen-fixing nodules in Phaseolus vulgaris.

## ANNOUNCEMENT

Rhizobium jaguaris CCGE525^T^ was isolated from nodules of the medicinal legume Calliandra grandiflora growing in a rain forest in Chiapas, Mexico, and was described as related to Rhizobium tropici group A (1). R. tropici group A was defined by 16S rRNA gene sequences and distinctive phenotypic characteristics (2). We report the genome sequence of strain CCGE525, the type strain of Rhizobium jaguaris.

A single colony from a freeze-dried culture sample of R. jaguaris CCGE525^T^ was incubated on peptone yeast (PY) medium (5 g/liter peptone, 3 g/liter yeast extract, and 0.6 g/liter CaCl_2_) for 3 days at 30°C. DNA was extracted from 3 ml of culture using a kit for cells and tissues (Roche Applied Science, Germany). A SMRTbell library of 15- to 20-kb insert size was constructed using standard protocols. The library was sequenced on a PacBio RS II sequencer (3) using P6-C4 chemistry, which yielded 3.4 Gb of data. Reads were filtered and assembled *de novo* using Canu v.1.5 (4). Annotation was performed using the NCBI Prokaryotic Genome Annotation Pipeline (https://www.ncbi.nlm.nih.gov/genome/annotation_prok/) (5). Amino acid sequences served as input to PhyloPhlAn (6) to predict evolutionary relationships. The progressive Mauve tool was used for genome alignments (7). DNA-DNA hybridization (DDH) values were computed using the Genome-to-Genome Distance Calculator v.2.1 (8). Average nucleotide identity (ANI) values were calculated as previously proposed (9) using the ANI calculator from the Konstantinidis Lab (http://enve-omics.ce.gatech.edu/ani/) (10). Default parameters were used for all programs.

The genome of R. jaguaris CCGE525^T^ (8,025,568 bp, 58.95% G+C content, and ∼278-fold coverage) consisted of a chromosome (4,575,315 bp), a chromid (2,584,926 bp), a symbiotic plasmid required for establishing interactions with legumes (550,563 bp), and an additional plasmid (314,764 bp). The genome coded for 8,400 predicted genes.

**FIG 1:**
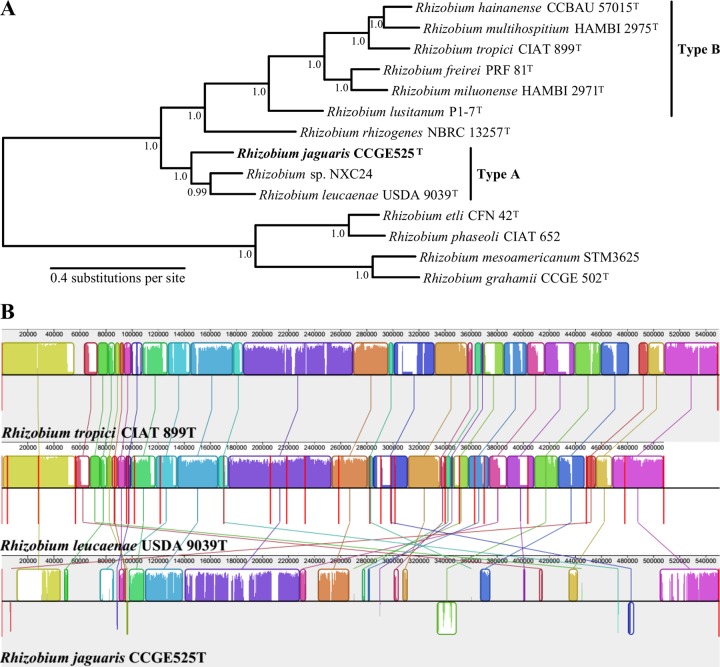
(A) Phylogenomic analysis showing the evolutionary relationships between sequenced *Rhizobium* strains belonging to the *R. tropici* type A and type B groups. The phylogeny is based on 373 marker proteins. Branch labels correspond to Shimodaira-Hasegawa-like support values. (B) Genome alignment of symbiotic plasmid sequences of the type strains *R. tropici* CIAT 899, *R. leucaenae* USDA 9039, and *R. jaguaris* CCGE525. Conserved regions are shown in colored blocks.

The R. tropici group A affiliation of R. jaguaris CCGE525^T^ was supported by its position in a genome tree (Fig. 1A). This phylogenomic approach increased resolution and confirmed the placement of R. jaguaris CCGE525^T^ as an isolated branch in the vicinity of Rhizobium leucaenae USDA 9039^T^.

R. jaguaris CCGE525^T^ is classified within the symbiovar calliandrae and has the capacity to form nitrogen-fixing nodules with common bean (1). A multiple sequence alignment revealed that the symbiotic plasmid of R. jaguaris CCGE525^T^ was less conserved and presented rearrangements compared to the similar symbiotic plasmids of Rhizobium tropici CIAT 899^T^ and R. leucaenae USDA 9039^T^ (Fig. 1B).

Sequence comparisons between the symbiotic plasmid of R. jaguaris CCGE525^T^ and the counterparts of R. tropici CIAT 899^T^ and R. leucaenae USDA 9039^T^ revealed ANI values of 85.40% and 85.48%, respectively. DDH estimates were 29.00% and 29.20% between the corresponding symbiotic plasmids. Thus, the symbiovar calliandrae is further validated.

Full-genome comparisons of R. jaguaris CCGE525^T^ revealed DDH estimates of 33.90% and 35.00% against R. leucaenae USDA 9039^T^ and *Rhizobium* sp. strain NXC24, respectively. ANI values of 87.07% and 87.50% were obtained when performing the same comparisons. These DDH and ANI values are below the thresholds for species boundaries of 70% and 95 to 96%, respectively (8, 9, 11–13). Thus, genome-based metrics allowed an accurate taxonomic circumscription of Rhizobium jaguaris.

### Data availability.

The genome sequence was deposited in GenBank under accession numbers CP032694 to CP032697. Raw sequences were submitted to the SRA database under accession number SRP174341.
